# Exploration of the correlation of serum polychlorinated biphenyl levels with luteal phase hormonal parameters and infertility in women with or without polycystic ovary syndrome

**DOI:** 10.3389/fendo.2023.1270949

**Published:** 2023-10-02

**Authors:** Alexandra E. Butler, Edwina Brennan, Daniel S. Drage, Thozhukat Sathyapalan, Stephen L. Atkin

**Affiliations:** ^1^ Research Department, Royal College of Surgeons in Ireland-Medical University of Bahrain, Busaiteen, Bahrain; ^2^ School of Medicine, Royal College of Surgeons in Ireland-Medical University of Bahrain, Busaiteen, Bahrain; ^3^ School of Geography, Earth and Environmental Sciences, University of Birmingham, Edgbaston, West Midlands, United Kingdom; ^4^ Queensland Alliance for Environmental Health Sciences, The University of Queensland, Woolloongabba, QLD, Australia; ^5^ Hull York Medical School, University of Hull, Hull, United Kingdom

**Keywords:** polychlorinated biphenyls, organic pollutants, pcos, infertility, *in vitro* fertilization, reproductive health

## Abstract

**Introduction:**

Polychlorinated biphenyls (PCBs) are organic lipophilic pollutants that accumulate in the body. Previous research has linked PCBs with menstrual function; therefore, this study was undertaken to investigate the correlation of PCBs with luteal phase hormonal parameters of menstrual function at day 21 in a group of non-obese women prior to *in vitro* fertilization (IVF).

**Methods:**

Fifty-eight non-obese Caucasian women from a UK academic center, 29 with polycystic ovary syndrome (PCOS) and 29 without, were recruited. PCOS women all had anovulatory infertility. Non-PCOS women: five with unexplained infertility, the remainder with male factor infertility (n=14) or tubal problems (n=10). Blood was withdrawn at day 21 of the menstrual cycle for non-PCOS women, at the time of mock embryo transfer. PCBs were measured using high resolution gas chromatography.

**Results:**

Only PCB118, PCB153, PCB138 and PCB180 were detected in all samples, and levels did not differ between PCOS and non-PCOS subjects. In non-PCOS subjects, PCB153, PCB138 and PCB180 inversely correlated with estradiol (p<0.05); PCB118 and PCB138 inversely correlated with follicle stimulating hormone (FSH) (p<0.05); PCB118 (p<0.05), PCB153, PCB138 and PCB180 (all p<0.01) inversely correlated with luteinizing hormone (LH). Control women without PCOS with unexplained infertility showed higher levels of PCB118, PCB153, PCB138 and PCB180 (p<0.05) compared to those control women without PCOS with tubal or male factor infertility, though other hormonal parameters did not differ other than that FSH that was lower in the unexplained group (p=0.01). The only correlation observed in PCOS women with anovulatory infertility was that between PCB180 and progesterone (p<0.05).

**Conclusion:**

PCBs correlated with luteal phase menstrual cycle hormones in control women without PCOS and may contribute to the mechanism of unexplained infertility; in PCOS women, no correlations of the PCBs were seen for estradiol, LH or FSH.

## Introduction

Polychlorinated biphenyls (PCBs) are organic pollutants that persist in the environment due to their resistance to biotransformation and high lipophilicity (1). Their accumulation is correlated to age (time of exposure) and obesity (2), with dietary consumption being the main route of exposure in humans (3). PCBs are classified as endocrine disrupters due to their observed thyroidogenic, estrogenic and antiandrogenic action (4) and are reported to affect the epigenome (5). Highly chlorinated PCB congeners (PCB118, PCB138, PCB153 and PCB180) reflect long term contamination, in contrast to PCB28, a low chlorinated volatile PCB that is degraded relatively fast and thus reflects relatively acute contamination (6). PCBs have been associated with infertility, higher levels associating with miscarriage (7). PCBs have been associated with endometriosis that causes pelvic pain and infertility (8); however, in a large cross-sectional study of self reported outcomes, whilst higher total PCB levels associated with fewer lifetime pregnancies, they did not correlate to the prevalence of infertility and pregnancy outcomes (9), though the infertility issues were not defined. Others report a health survey that suggested a prolonged time to pregnancy and reduced fertility in women previously exposed to PCBs (10); however, no study has looked at correlations between PCBs and causes of infertility in a defined population.

Polycystic ovary syndrome (PCOS), associated with menstrual dysfunction, infertility, hirsutism, acne, obesity and metabolic syndrome (11), is the most common endocrine disorder among women of reproductive age, with a reported prevalence of 6-10% (12). PCOS is a proinflammatory state with increased insulin resistance and elevation in the inflammatory marker C-reactive protein (CRP) (13), and is a complex multigenetic heterogenous disorder with evidence of epigenetic and environmental influences resulting in varied phenotypes, clinical manifestations and metabolic consequences (14).

We hypothesised that PCBs would be unlikely to be associated with infertility causes, but may relate to hormone levels in the luteal phase of the menstrual cycle; therefore, this pilot study was undertaken in two cohorts of women preparing to undergo IVF: firstly, women with PCOS and anovulatory infertility and, secondly, control women without PCOS in whom the luteal phase was carefully defined at the time of mock embryo transfer, and in whom the hypothalamo-ovarian axis would have been considered to be normal given the causes of infertility being male factor infertility, tubal problems or unexplained infertility.

## Materials and methods

The study design was a case-control study. Participants were sequentially recruited in 2015 from the Hull *In Vitro* Fertilization (IVF) Unit, UK, following ethical approval from The Yorkshire and The Humber NRES ethical committee, UK (approval number 02/03/043). Participants with known immunological disease, diabetes, renal or liver insufficiency, acute or chronic infections, inflammatory disease, age <20 or >45 years, body mass index (BMI) >30kg/m^2^ and those not undergoing IVF treatment, were excluded from the study. PCOS was diagnosed using the revised 2003 Rotterdam criteria (15). All PCOS subjects had hypogonadotropic anovulatory infertility (n=29; mean age 30.9 ± 4.8 years, mean weight 72.5 ± 13.1 kg, mean BMI 26.0 ± 3.8 kg/m^2^), whilst for the non-PCOS women the cause of infertility was determined to be male factor infertility (MFI, n=14), tubal problems (n=10) [MFI and tubal problems were combined as explained infertility (n=24; mean age 32.5 ± 4.5 years, mean weight 67.2 ± 12.0 kg, mean BMI 24.9 ± 3.5 kg/m^2^)] or unexplained infertility (n=5; mean age 34.7 ± 3.3 years, mean weight 71.5 ± 12.9 kg, mean BMI 27.1 ± 3.6 kg/m^2^). Of the 58 participants recruited (29 PCOS and 29 controls), written informed consent was obtained from all participants (16). No subject had been on any prescribed or over-the-counter medication, all were non-smokers and none consumed alcohol in the preceding six months, data that was collected in the full medical history.

### Sample collection

At 21 days of the menstrual cycle, prior to IVF treatment, when no hormonal treatment had been given, mock embryo transfer was undertaken as part of normal clinical practice, when ovarian and endometrial ultrasound were also performed. Single fasting blood samples were taken from each subject at the time of mock embryo transfer using vacutainers (Becton Dickinson, New Jersey, USA): ethylenediaminetetraacetic acid (EDTA) for glycosylated hemoglobin A1c (HbA1c) measurement in whole blood; sodium fluoride for glucose measurement; no additive for serum measurement of all other parameters. Serum was prepared by centrifugation at 3500×g for 15 min at 4°C and stored at −80°C. Fasting blood glucose (FBG) was measured using a Synchron LX20 analyzer (Beckman-Coulter, High Wycombe, UK). Serum insulin was measured by competitive chemiluminescent immunoassay (DPC Immulite 2000 analyzer, Euro/DPC, Llanberis, UK). Homeostatic model assessment for insulin resistance (HOMA-IR) was calculated using the formula ((Insulin x glucose)/22.5) (17). Serum follicle stimulating hormone (FSH), luteinizing hormone (LH), estradiol and progesterone were measured using a chemiluminescent microparticle immunoassay technology (Abbott Diagnostics, Maidenhead, UK). C-reactive protein (CRP), total cholesterol (TC) and triglycerides (TG) were measured enzymatically (Synchon LX20 analyzer, Beckman-Coulter, High Wycombe, UK). Total serum lipid (TSL) was determined using the formula ((2.27 x TC) + TG + 62.3 mg/dL) (18). Anti-müllerian hormone (AMH) was measured using an immunoenzymatic assay (Beckman-Coulter, High Wycombe, UK). HbA1c was measured using ion-exchange chromatography. Estimated glomerular filtration rate (eGFR) was calculated using the formula (175 x (SCr, mg/dL)-1.154 x (age, years)-0.203 x 0.742) (19). Androgens were measured by liquid chromatography tandem mass spectrometry (LC/MS/MS; Acquity UPLC-Quattro Premier XE-MS, Waters, Manchester, UK). An immunometric assay with fluorescence detection (DPC Immulite 2000 analyzer, Siemens, Camberley, UK; upper limit 2.0 nmol/l) was used to measure sex hormone binding globulin (SHBG). The formula ((testosterone/SHBG) x 100) was used to calculate free androgen index (FAI). An Abbott Architect i4000 immunoassay analyzer (Abbott Diagnostics Division, Maidenhead, UK) was used to measure thyroid hormone levels.

### Polychlorinated biphenyl analysis

Samples were analyzed for 7 indicator PCBs: PCB28, PCB52, PCB101, PCB118 (a dioxin like PCB), PCB138, PCB153 and PCB180. 5mL of serum was spiked with 5ng of each of 13C12-labelled PCBs (Wellington Laboratories, Guelph, Ontario, Canada) in 50mL Falcon tubes. Extraction and clean-up were performed using a previously described protocol (20). Clean extracts were evaporated to near-dryness, reconstituted in 50µL hexane containing 2.5 ng 13C12-PCB-141 as a recovery standard, and transferred to inserted autosampler vials prior to analysis. PCBs were determined using high resolution gas chromatography (Thermofisher TRACE 1300, Loughborough, UK) coupled with high resolution mass spectrometry (HRGC/HRMS, Thermofisher DFS, Loughborough, UK) with quality assurance checks using previously described methods (21). A sum PCB (∑PCB) variable was calculated by adding the molar concentrations of PCB congeners analyzed.

### Statistical analysis

There are no published studies to base a power calculation on; therefore, this was an exploratory pilot study on which a power calculation could be determined based on the results observed. Descriptive data are presented as mean ± standard deviation (SD) for continuous data. Serum PCB concentrations are expressed as geometric mean ± SD. PCB levels, metabolic outcomes and hormone concentrations were assessed for normality and Independent T or Mann-Whitney U tests were used to compare means/medians, as appropriate. Potential correlations between PCBs and metabolic and fertility parameters were examined using exploratory Pearson’s correlations. A p-value of <0.05 was considered indicative of statistical significance. Statistical analysis was carried out using Graphpad Prism version 9.5.1 (San Diego, CA, USA).

## Results

### Demographics, metabolic outcomes, and hormone levels

PCOS and non-PCOS women did not differ in age, BMI, insulin, HOMA-IR or TSL (**Table 1**), nor were there any differences in these variables for unexplained, tubal and anovulatory infertility. PCOS women had higher FAI (3.1 ± 2.9 vs 1.7 ± 3.4, p=0.002), testosterone (1.1 ± 0.5 vs 0.8 ± 0.4 nmol/L, p=0.01), androstenedione (4.1 ± 1.5 vs 2.5 ± 1.2 nmol/L, p<0.001), AMH (56 ± 14 vs 24 ± 14 ng/mL, p<0.001), LH (14.9 ± 14.3 vs 6.0 ± 8.3 iU/L, p=0.01) and triglycerides (1.3 ± 0.7 vs 1.0 ± 0.5 mmol/L, p=0.03) compared to control women without PCOS. FBG and SHBG were lower in PCOS women (4.5 ± 0.8 vs 4.8 ± 0.3 nmol/L, p=0.04 and 65 ± 51 vs 113 ± 83 nmol/L, p=0.008, respectively) (22).

**Table 1:** Demographic data, together with metabolic and hormone level measurements for women with (n=29) and without (n=29) PCOS.

	Control women without PCOS (n=29)		Women with PCOS (n=29)		
	Mean	SD	Mean	SD	p
Age (Years)	33	4	31	5	0.10
BMI (kg/m^2^)	25.4	3.6	26.0	3.8	0.49
Insulin (µIU/ml)	7.6	4.1	8.1	4.7	0.94
HOMA-IR	1.7	1.0	2.0	1.6	0.96
FBG (nmol/L)	4.8	0.3	4.5	0.8	0.04
TSL (mg/dL)	573	106	583	134	0.77
AMH (ng/mL)	24	14	56	14	< 0.001
eGFR (mL/min/1.73 m^2^)	96	17	88.1	10.3	0.06
CRP (mg/L)	2.5	2.3	2.7	2.6	0.99
HbA1c (mmol/mol)	30.7	6.2	32.0	3.3	0.72
FAI	1.7	3.4	3.1	2.9	0.002
Testosterone (nmol/L)	0.8	0.4	1.1	0.5	0.01
SHBG (nmol/L)	113	83	65	51	0.008
Androstenedione (nmol/L)	2.5	1.2	4.1	1.5	< 0.001
TSH (mU/L)	2.2	1.1	2.6	3.0	0.86
Free-T3 (pmol/L)	4.8	0.7	4.8	0.7	0.9
Free-T4 (pmol/L)	11.2	1.3	11.5	2.2	0.61
Estradiol (pmol/L)	457	301	431	463	0.84
Progesterone (nmol/L)	15.0	15.4	7.1	9.1	0.08
FSH (iU/L)	3.3	2.2	4.1	2.3	0.18
LH (iU/L)	6.0	8.3	14.9	14.3	0.01
Cholesterol (mmol/L)	4.8	0.8	4.7	1.1	0.65
Triglycerides (mmol/L)	1.0	0.5	1.3	0.7	0.03

BMI, Body mass index; HOMA-IR, homeostatic model assessment for insulin resistance; FBG, fasting blood glucose; TSL, total serum lipids; AMH, anti-müllerian hormone; eGFR, estimated glomerular filtration rate; CRP, C-reactive protein; HbA1c, glycosylated hemoglobin A1c; FAI, free androgen index; SHBG, sex hormone binding globulin; TSH, thyroid stimulating hormone; Free-T3, free-triiodothyronine; Free-T4, free-thyroxine; FSH, follicle stimulating hormone; LH, luteinizing hormone; PCOS, polycystic ovary syndrome; iU, international units; SD, standard deviation; sample size (n); significance at p=0.05 (p).

### Serum PCB levels, and correlations with metabolic outcomes and hormone levels

Geometric mean (GM) concentrations of frequently detected PCBs and the ∑PCB variable did not differ between PCOS and non-PCOS women (p=ns), as previously reported in this study cohort (23); non-PCOS versus PCOS (ng/g lipid): PCB118 (5.1 ± 1.4 vs 5.4 ± 1.5), PCB138 (11.6 ± 1.5 vs 10.1 ± 1.7), PCB153 (15.0 ± 1.6 vs 12.2 ± 1.8), PCB180 (13.5 ± 1.6 vs 10.7 ± 1.7), ∑PCBs (48.3 ± 1.5 vs 41.1 ± 1.6) (23). As reported before, using multivariable linear regression there were no associations between PCBs and blood glucose, HOMA-IR, insulin or HbA1c, nor with FAI, androstenedione or testosterone (23).

Gonadal hormones: in the control women without PCOS, PCB153, PCB138 and PCB180 inversely correlated with estradiol (p<0.05); there was no correlation between PCBs and progesterone in control women without PCOS.

**Figure 1:**
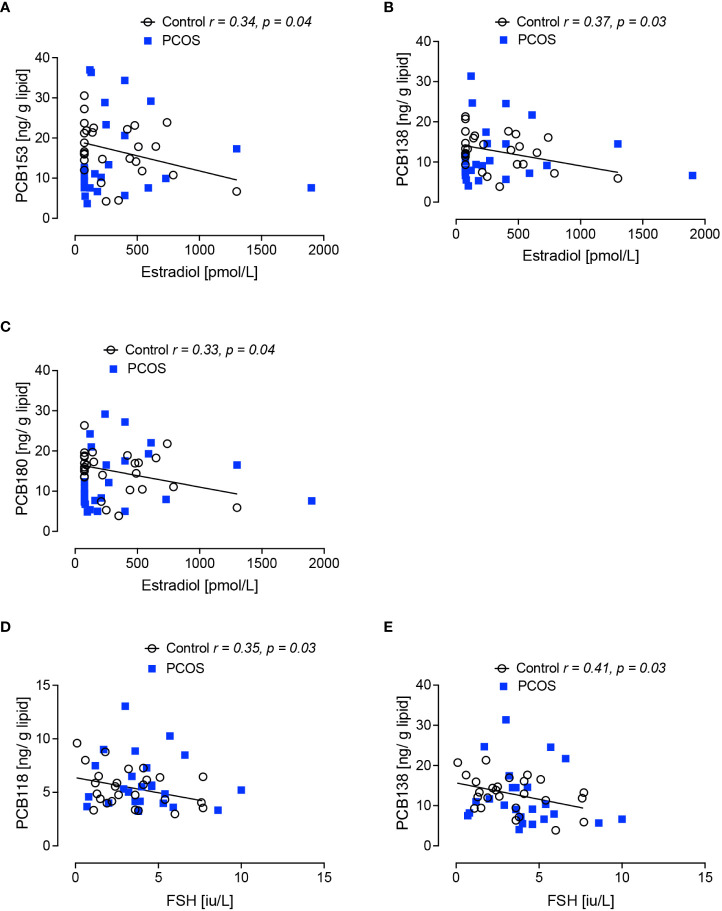
Correlations of serum concentrations of PCBs with serum estradiol and follicle stimulating hormone (FSH) levels in control women without polycystic ovary syndrome at 21 days of the menstrual cycle. Negative correlations of estradiol with PCB153 **(A)**, PCB 138 **(B)** and PCB180 **(C)**, and of follicle stimulating hormone (FSH) with PCB118 **(D)** and PCB138 **(E)** were found in women without polycystic ovary syndrome (black circles) but not in women with polycystic ovary syndrome (PCOS, blue squares).

**Figure 2:**
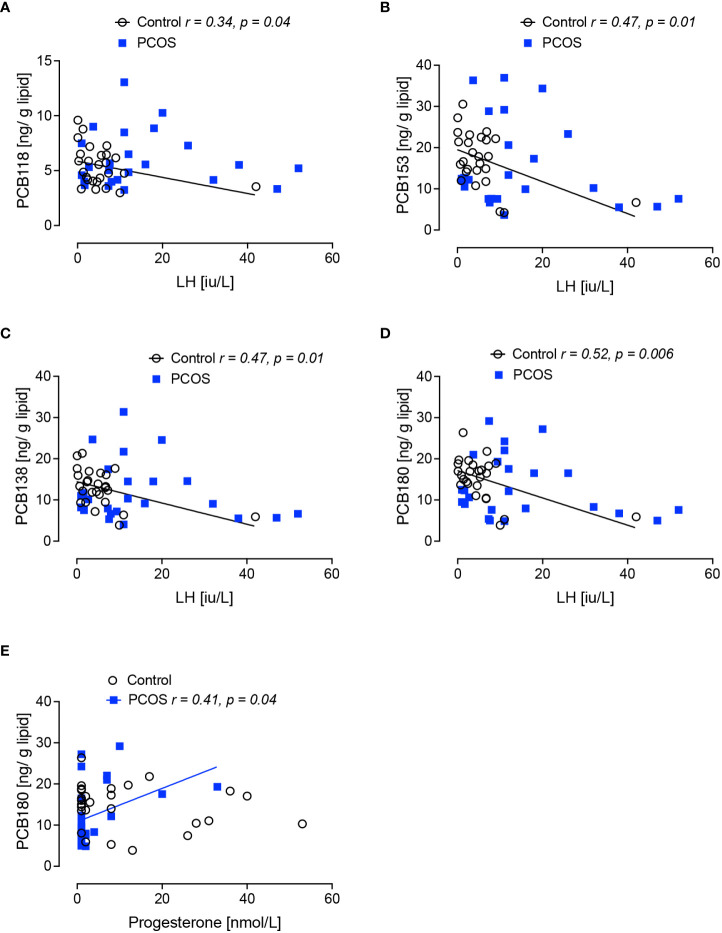
Correlations of serum concentrations of PCBs with serum lutenizing hormone (LH) and progesterone levels in women with polycystic ovary syndrome and control women without polycystic ovary syndrome at 21 days of the menstrual cycle. Negative correlations of lutenizing hormone (LH) with PCB118 **(A)**, PCB 153 **(B)**, PCB138 **(C)** and PCB180 **(D)** were found in women without polycystic ovary syndrome (black circles) but not in women with polycystic ovary syndrome (PCOS, blue squares). A positive correlation of progesterone with PCB180 **(E)** was found in women with PCOS but not in control women without PCOS.

Pituitary hormones: in the control women without PCOS, PCB118 and PCB138 inversely correlated with FSH (p<0.05); PCB118, PCB153, PCB138 and PCB180 inversely correlated with LH (p<0.05, p<0.01, p<0.01 and p<0.01, respectively) (**Figures 1**, **2**).

**Figure 3:**
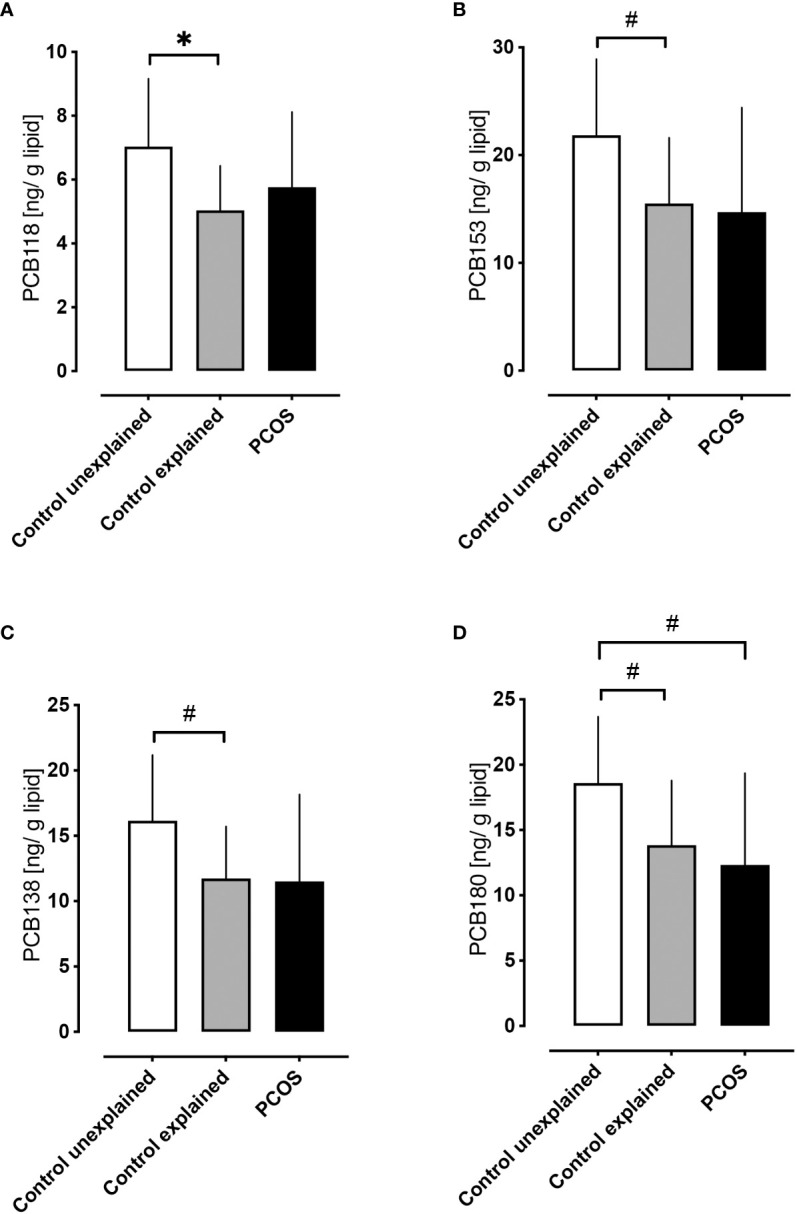
A comparison of serum concentrations of PCBs in control women without polycystic ovary syndrome with unexplained infertility versus control women without polycystic ovary syndrome with explained infertility (both at 21 days of menstrual cycle) versus women with PCOS. Higher levels of PCB118 **(A)**, PCB153 **(B)**, PCB138 **(C)** and PCB180 **(D)** were found in control women without PCOS with unexplained infertility (n=5) (white bars) versus control women without PCOS with explained fertility [due to tubal (n=10) or male factor infertility (n=14); gray bars]. Levels of PCB118 **(A)**, PCB153 **(B)** and PCB138 **(C)** in women with PCOS (n=29; black bars) did not differ from control women with either explained or unexplained infertility; only PCB180 **(D)** differed in women with PCOS versus control women without PCOS with unexplained infertility *p<0.01, ^#^p<0.05.

Control women without PCOS with unexplained infertility showed higher levels of PCB118, PCB153, PCB138 and PCB180 (p<0.05) compared to those control women without PCOS with tubal or male factor infertility (**Figure 3**), though other hormonal parameters did not differ between infertility causes, other than FSH that was lower in the unexplained group (p=0.01).

In women with PCOS, no correlations of the PCBs were seen for estradiol, LH or FSH. The only correlation observed in PCOS with anovulatory infertility was a positive correlation between PCB180 and progesterone (p=0.04) (**Figure 2E**).

## Discussion

This study suggests that PCB concentrations may affect hormones of the luteal phase of the menstrual cycle that may then be reflected in infertility. It is recognized that PCBs and other endocrine disruptors may affect thyroid function and the actions of both estrogens and androgens (4). PCBs have been associated with infertility with a reported association with the development of endometriosis (8), and reduced fertility suggested by fewer lifetime pregnancies and prolonged time to pregnancy after PCB exposure (9, 10), but their relationship to individual causes of infertility has not previously been examined. In addition, PCBs have been linked to menstrual cycle irregularities, early menopause and miscarriage (7, 24), though that the PCBs are associated with effects on the menstrual cycle hormones is less reported. This study suggests that PCBs may be associated with the hormones of the menstrual cycle in the mid luteal phase, specifically FSH, LH and estradiol, but only in the women without PCOS. This is not surprising as, if PCBs are affecting the menstrual cycle, then anovulatory women with PCOS in this cohort who were not experiencing a menstrual cycle at the time of the study would not show any effect of their action unless the menstrual cycle is induced. Therefore, the correlations between PCBs (specifically PCB118, PCB153, PCB138 and PCB180) with the mid luteal phase only occurred in women exhibiting a regular menstrual cycle: PCB153, PCB138, PCB180 inversely correlated with estradiol, PCB118 and PCB138 inversely correlated with FSH; PCB118, PCB153, PCB138 and PCB180 inversely correlated with LH. Taken together, this suggests that the PCBs may act at the level of the hypothalamus (25) with an effect on the gonadotrophin releasing hormone pulse generator affecting LH, and potentially also at the level of the pituitary (25), with the correlation between estradiol and PCB being a secondary effect; thus, PCB180 in particular (26) may affect both LH and FSH secretion, thereby affecting estradiol. However, it cannot be excluded that PCBs may potentially also be acting at the level of the ovary and there is evidence of PCBs affecting follicular ovarian reserve (27) and follicular steroidogenesis (28). This suggests that PCB-induced perturbation of the menstrual hormones may contribute and potentially be additive to other factors causing infertility, and that PCBs are not related to anovulation as a mechanism for how endocrine disrupting chemicals may affect fertility.

Control women without PCOS with unexplained infertility showed higher levels of PCB118, PCB153, PCB138 and PCB180 compared to those control women without PCOS with tubal or male factor infertility, though other hormonal parameters did not differ other than FSH that was lower in the unexplained group. There are many etiologies for “unexplained” infertility and the contribution of PCBs is just one of many, including ovulatory dysfunction, sperm quality and quantity, fallopian tube function, endometriosis and immune factors (29). The possibility that the PCB association is an epiphenomenon for another contributory factor(s) must also be considered. There are several endocrine disruptor groups that have been associated with infertility, such as organochloride pesticides, heavy metals and dioxins (30); consequently, it may be that a combination of endocrine disrupting chemicals such as organochloride pesticides and polybrominated diethyl ethers among others (30) act in concert to affect fertility. The reported association of patient age and weight with PCB levels (31) could suggest that the PCBs are just a marker of another underlying process such as obesity. Clearly, this was not the case in this study, where all of the subjects were non-obese and not insulin resistant, thus suggesting that PCBs may have a more direct role than previously realized or represent a biological marker for unexplained infertility. With PCBs affecting hypothalamo-pituitary function resulting in effects on the menstrual cycle, this would suggest that in the evidence-based treatment of unexplained infertility (32) that early medical intervention rather than expectant management may be warranted in those with documented elevation of PCB levels.

The strengths of this study lie in the study design of differing causes of infertility in non-obese women with PCOS and control women without PCOS matched for age and BMI, together with the measurement of relevant metabolic parameters and hormone levels. In addition, as these women were undergoing an IVF program for fertility, none had been on any hormonal contraception and they had all stopped any alcohol consumption, thus removing these confounders from the analysis. The limitations of the study include the small sample size and the potential lack of generalizability to ethnicities other than a Caucasian population. Comparison of the differing causes of fertility within the control group without PCOS needs to be interpreted with care as there were only 5 cases of unexplained infertility versus 24 in the explained fertility group. There is a need to determine the association of the endocrine disruptors to the menstrual cycle to ascertain causality and to perform a larger robust study incorporating the differing causes of infertility to determine the relationship to the differing endocrine disrupting chemicals. Of significance, much of the literature to date is based on animal studies that may not reflect what occurs in humans. It is likely that any contribution of the PCB effect on infertility may be multifactorial, possibly acting in combination with other endocrine disruptor chemicals, or contribute to pathophysiological processes such as obesity.

In conclusion, PCBs, specifically PCB118, PCB153, PCB138 and PCB180, are correlated with luteal phase menstrual cycle hormones in women without polycystic ovary syndrome. The serum levels of these PCBs were elevated in control women without PCOS with unexplained infertility versus control women without PCOS with explained infertility and thus may contribute to the mechanism of unexplained infertility, though larger robust studies are needed to confirm these findings and to ascertain causality.

## Data availability statement

The raw data supporting the conclusions of this article will be made available by the authors, without undue reservation.

## Ethics statement

The studies involving humans were approved by The Yorkshire and The Humber NRES ethical committee, UK. The studies were conducted in accordance with the local legislation and institutional requirements. The participants provided their written informed consent to participate in this study.

## Author contributions

AB: Conceptualization, Formal Analysis, Visualization, Writing – original draft, Writing – review & editing. EB: Writing – original draft, Writing – review & editing. DD: Investigation, Writing – review & editing. TS: Conceptualization, Methodology, Writing – review & editing. SA: Conceptualization, Methodology, Validation, Visualization, Writing – original draft, Writing – review & editing.
